# Different interventions for the treatment of patent ductus arteriosus in children: a protocol for a network meta-analysis

**DOI:** 10.1186/s13643-023-02195-4

**Published:** 2023-03-02

**Authors:** Xin Zhang, Xiao-Dong Hou, Wen-Xin Wang, Kang Yi, Xin-Kuan Wang, Fan Ding, Xin-Xin Li, Tao You

**Affiliations:** 1 The First School of Clinical Medicine of Gansu University of Chinese Medicine, Lanzhou, China; 2 Gansu International Scientific and Technological Cooperation Base of Diagnosis and Treatment of Congenital Heart Disease, Lanzhou, China; 3 Department of Cardiovascular Surgery, Gansu Provincial Hospital, Lanzhou, China

## Abstract

**Introduction:**

Patent ductus arteriosus (PDA) is one of the most common congenital heart diseases. Once the PDA is diagnosed, it needs to be dealt with in time. At present, main methods include pharmacological treatment, surgical closure, and interventional closure for treatment of PDA. However, the effect of different interventions in PDA management is still controversial. Thus, our study aims to assess the effectiveness of different interventions together and estimate the sequence of these therapies for PDA children. Meanwhile, it is necessary to conduct a Bayesian network meta-analysis to compare the safety of different interventions comprehensively.

**Methods and analysis:**

To the best of our knowledge, this is the first Bayesian network meta-analysis comparing the efficacy and safety of different interventions for the treatment of PDA. PubMed, Embase, Cochrane Library, Web of Science, gray literature, and trial registry databases were searched from inception to December 2022. We will extract and report data according to methodological guidelines for Bayesian network meta-analysis by the Preferred Reporting Items for Systematic Reviews and Meta-Analyses Protocols (PRISMA-P). Primary PDA closure, overall PDA closure, technical success, surgical success rate, mortality during hospital stay, operation time, intensive care unit stay, intraoperative radiation dose, radiation exposure time, total postoperative complication rate, and postoperative major complication rate will be defined as the outcomes. The quality of all random studies will be assessed using ROB, and quality of evidence for all outcomes will be judged by using the Grading of Recommendations Assessment, Development and Evaluation (GRADE).

**Ethics and dissemination:**

The results will be disseminated through peer-reviewed publication. Since no private and confidential patient data will be contained in the reporting, there are no ethical considerations associated with this protocol.

**Systematic review registration:**

INPLASY2020110067.

**Supplementary Information:**

The online version contains supplementary material available at 10.1186/s13643-023-02195-4.

## Strengths and limitations of this study

- This paper will be the first study to compare the efficacy and safety of surgical procedures for PDA patients using Bayesian network meta-analysis.
- We will use the Grading of Recommendations Assessment, Development and Evaluation (GRADE) approach to evaluate the quality of evidence.
- The results of this study will help surgeons and patients to select appropriate treatments.
- Our results will be limited by both the quantity and quality of the trials available for review.

## Introduction

Congenital heart disease (CHD) is the most prevalent human birth malformation with an incidence of one approximately in 100 newborns. CHD is also a major cause of perinatal and infant mortality, with about 220,000 deaths worldwide each year [1]. Ductus arteriosus is a vascular structure connecting the junction of the main and left pulmonary artery to the descending aorta just distal to the origin of left subclavian artery in fetal life and forms an important outflow conduit for right ventricular output to bypass the high resistance pulmonary arterial circulation. The duct is functionally closed 12 to 18 h after birth and anatomically in 2 to 3 weeks [2–4]. If it is not closed beyond 3 months of life in full-term infants and beyond 1 year in premature infants, it is called patent ductus arteriosus (PDA) [2]. It is reported incidence rate of PDA in term neonates is one in 2000 births, approximately 5%-10% of all congenital heart disease [3]. The natural history of PDA is variable, including asymptomatic, incidentally detected defect to congestive heart failure, atrial arrhythmias, endocarditis, ductal aneurysm, pulmonary vascular disease, and Eisenmenger’s syndrome [5]. Therefore, once the PDA is diagnosed, it needs to be dealt with in time. However, different treatment methods have various effects on patients with PDA. At present, traditional treatment includes pharmacological treatment, surgical closure, and interventional closure [6].

Currently, three pharmacological agents—ibuprofen, acetaminophen, and indomethacin—are used for PDA closure in preterm infants [7]. Indomethacin started to be used in 1970. It was first trialed for the treatment of infants with symptomatic PDA [8]. The first trial that confirmed indomethacin’s efficacy for PDA management was published in 1983 [9]. However, it has been associated with several adverse events, such as non-intracranial hemorrhage and gastrointestinal (GI) bleeding, weakened platelet aggregation, and renal failure. In 2003, ibuprofen was confirmed that it may be as effective in PDA closure as indomethacin by Ohlsson et al. [10], with the potential benefits of fewer GI and renal side effects. In 2011, Hammerman et al. [11] suggested the use of acetaminophen as another treatment option in preterm infants with PDA, who had failed or had contraindications to indomethacin and ibuprofen. Meanwhile, at the same time, pharmacological treatment is controversial in the primary efficacy and safety aspects, particularly in premature infants [12]. Therefore, pharmacological treatment has certain limitations. Surgery closure of PDA has been shown to be safe [6]. There are many surgical methods can be used to treat PDA. Thoracotomy has always been a standard method for treating PDA. In 1938, Robert Edward Gross successfully ligated a persistent ductus arteriosus [13, 14]. Traditionally, surgical ligation has been used to provide definitive ductal closure, but surgery has risks, including pneumothorax, hypothermia, bleeding, phrenic nerve palsy, wound infection, vocal cord paralysis, and thoracic scoliosis [15–17]. In order to minimize these risks, a less invasive surgical approach can be used, derived from the technique by mini-thoracotomy [17, 18]. Mini-thoracotomy is a simple technique and provides the closest approach to the PDA when compared to the other thoracotomy incisions. The mini-thoracotomy do not use any costal retractor, only finger retractor for exposure, and only little traction on the apex of the left lung as compared with the other techniques, which results in less risk of surgical complications and hemorrhage [19]. With the development of surgical techniques, the video-assisted thoracoscopic surgery (VATS) repair PDA was first described by Laborde and colleagues in 1993 [20]. Studies have shown that the VATS approach is associated with decreased operative time, smaller incisions with less postoperative pain, earlier extubation, and decreased hospital and intensive care unit (ICU) stay compared with the open approach [21, 22]. Transcatheter PDA closure first was achieved in 1967 by Porstmann [23]. In the past decades, transcatheter closure has become the primary approach to closure of most PDA. The original transcatheter PDA closure techniques utilized large arterial and venous delivery sheaths [17]. Meanwhile, the method usually requires joint monitoring and guidance of fluoroscopy. However, intraoperative fluoroscopy radiation damages both the surgeon and the patient, and angiography may cause renal failure in patients [24]. Evan M. et al. developed a technique, which combines the attributes of fluoroscopy and echocardiography [16]. This technique used a transfemoral venous and arterial approach without the use of angiography. In 2011, Bentham et al. reported a novel retrograde femoral arterial transthoracic echocardiography (TTE)-guided technique of PDA closure. Unfortunately, this approach utilizes femoral arterial access without fluoroscopy and placed infants of PDA at risk for limb ischemia [16, 25]. Recently, a study suggested that PDA occlusion using a transfemoral venous approach under TTE guidance is technically feasible and safe [26].

At present, some pairwise meta-analyses have compared the efficacy and safety of some procedures. Network meta-analysis (NMA) can estimate the relative effectiveness of all interventions and the sequence of interventions, even in the absence of a head-to-head comparison of all interested interventions [26]. Thus, our study aims to assess the relative effectiveness of different therapies together and estimate the sequence of these therapies for PDA children. Meanwhile, it is necessary to conduct a NMA to compare the effects of different interventions comprehensively.

## Methods

Bayesian network meta-analysis will be carried out. This protocol is reported according to the Preferred Reporting Items for Systematic Review and Meta-Analysis Protocols (PRISMA-P) [27], and the checklist is presented in online Supplementary Appendix 1. This network meta-analysis will be reported in accordance with PRISMA extension version (PRISMA-NMA) [28]. This study protocol has been registered on the international prospective register of systematic review (INPLASY2020110067).

### Search strategy

We will perform electronic search until December 2022 in the following databases: PubMed, Embase, Cochrane Library, and Web of Science. We will also search ClinicalTrials.gov, WHO International Clinical Trials Registry Platform (ICTRP), and International Standard Randomized Controlled Trial Number Registry for all registered clinical trials and randomized controlled trials. Furthermore, we will search the gray literature in Google Scholar, OpenGrey, and ProQuest Dissertation. The reference lists of articles and relevant systematic reviews will be tracked to identify other relevant studies. No restrictions on language will be set. If we find more relevant key words during our primary search, we will modify the search syntax. All searches will be updated before submitting the review.

The PubMed search strategies are as follows:

- #1 “Ductus Arteriosus, Patent” [MeSH Terms]
- #2 “patent ductus arteriosus” [Title/Abstract]
- #3 #1 OR #2
- #4 "Thoracic Surgery, Video-Assisted "[MeSH Terms] OR "video-assisted thoracoscopic

surgery"[Title/Abstract] OR "Minimally Invasive Surgical Procedures"[MeSH Terms] OR "minimally invasive"[Title/Abstract] OR "Minimal Access Surgical Procedures"[Title/Abstract] OR "mini-invasive"[Title/Abstract] OR "minimal surgical procedure*"[Title/Abstract] OR "Surgical ligation"[Title/Abstract] OR "surgical closure"[Title/Abstract] OR "Transthoracic"[Title/Abstract] OR "transcatheter"[Title/Abstract] OR "percutaneous closure"[Title/Abstract] OR "pharmacological"[Title/Abstract] OR "Cyclooxygenase Inhibitors"[MeSH Terms] OR "acetaminophen"[MeSH Terms] OR "COXI"[Title/Abstract] OR "Indomethacin"[Title/Abstract] OR "indometacin"[Title/Abstract] OR "indocid"[Title/Abstract] OR "ibuprofen"[Title/Abstract] OR "brufen"[Title/Abstract] OR "motrin"[Title/Abstract] OR "paracetamol"[Title/Abstract]. #5 #3 AND #4

### Eligibility and exclusion criteria

#### Eligibility criteria

1) Types of study: We will include randomized controlled trials. The number of cases included in each group must be greater than ten. Retrospective cohort studies, prospective cohort studies, case–control studies, and case series will not be included.

2) Types of participants: PDA children were confirmed clinically and by transthoracic echocardiography and scheduled for pharmacological treatment, surgical closure of PDA, or interventional closure. There were no restrictions in children weight and age. PDA diameter ranged from 2 to 5.5 mm.

3) Types of interventions: Pharmacological treatment (acetaminophen, indomethacin, or ibuprofen), mini-thoracotomy, VATS, fluoroscopy-guided transfemoral venin and artery of percutaneous closure, fluoroscopy combined with TTE-guided transfemoral venin and artery of percutaneous closure, TTE-guided transfemoral artery of percutaneous closure, and TTE-guided transfemoral vein of percutaneous closure.

4) Types of comparison groups: Surgical ligation for surgical interventions and placebo for pharmacological treatment. If there are head-to-head studies, these studies which compare surgical ligation with other mini-invasive treatments, treatment between different medicines, or pharmacological with surgical treatments will be included.

5) Types of outcomes.

The primary outcomes include the following: primary PDA closure (defined as echocardiography-confirmed closure of PDA after the first course of the treatment), overall PDA closure (defined as echocardiography-confirmed closure of PDA after one or more courses of the treatment), and technical success (defined as patient leaving the procedure room (catheterization laboratory or appropriate alternative) with a device or coil in the PDA. Cases were also considered successful in the event of coil/device embolization that was retrieved during the same procedure, and the PDA was closed with a different size of device/coil.)

The secondary outcomes include the following: mean days/hours needed for closure of PDA, surgical success rate, mortality during hospital stay, operation time, intraoperative radiation dose, radiation exposure time, total postoperative complication rate, postoperative major complication rate, intensive care unit stay, total hospital stays, postoperative hospital stays, and total cost.

#### Exclusion criteria

1. Children were diagnosed with other congenital heart diseases (such as ventricular septal defect or atrial septal defect).
2. Those studies which assessed the change of cardiac function and cardiac physiology changes after PDA treatment
3. No treatment (conservative management)

#### Selection of studies

Literature search records will be imported into EndNote X8.1 (Thomson Reuters (Scientific) LLC Philadelphia, PA, USA) software. First, the EndNote X8.1 will be used to exclude duplicate articles, and then, two reviewers will independently screen the titles and abstracts and exclude the articles that do not fulfill the inclusion criteria. If it is hard to judge, full texts will be retrieved. After then, two reviewers will cross-check whether the final selected research is consistent. If there are any disagreements between two reviewers during the selection of eligible studies, a third reviewer will involve resolving the disagreements.

#### Data extraction

A standard data abstraction form will be created using Microsoft Excel 2019 (Microsoft, Redmond, WA, USA, www.microsoft.com). Then, two independent reviewers will extract the basic characteristics and the data of outcomes. The extracted data will include the following: authors, gender, age, birthweight, year of publication, study design, inclusion criteria, medicine dosing protocol, sample size, primary closure rate, reopening rate, mortality, intraventricular hemorrhage (IVH all grades), necrotizing enterocolitis (NEC all grades), pulmonary hemorrhage (blood-stained liquid flowing from the trachea of the infant), bronchopulmonary dysplasia, retinopathy of prematurity, renal dysfunction, hepatic dysfunction, gastrointestinal bleeding, treatment methods, device used, median follow-up, PDA diameter, etc. If there is a discrepancy between the two reviewers, a third researcher will be consulted. We will randomly select five to ten studies to check the completeness of the data abstraction form. The data abstraction form will be complemented by the pilot trial. We will contact the corresponding author to query information when the essential data are insufficient or missing in the original study through sending an email. Studies will be excluded if we are unable to get access to the data, and the reasons for exclusion will be reported in detail.

#### Risk-of-bias assessment

Randomized controlled trials will be assessed using the Cochrane Handbook’s Risk of Bias (ROB) assessment tool [29]. Studies will be reviewed and scored as “high risk,” “low risk,” or “unclear” in each of the following domains: random sequence generation, allocation concealment, blinding of participants and personnel, blinding of outcome assessment, incomplete outcome data, selective reporting, and other bias. Inter-rater reliability of the ROB tool has been demonstrated to range from fair to substantial depending on the assessment domain [24]. The risk of bias will be assessed by two independent reviewers, and conflicts will be resolved by a third reviewer.

#### Geometry of the network

A network plot will be created to describe and present the geometry of the treatment network of comparisons across trials using STATA (16.0; Stata Corporation, College Station, TX, USA). If the trial is not linked by treatments, we will exclude it from network meta-analysis and just describe the findings of the study.

In the network plot, nodes represent different interventions, and edges represent a head-to-head comparison between interventions. The size of the nodes and the thickness of the edges are associated with the sample size of the intervention and the number of trials included, respectively.

For pharmacological treatment, a network will be created. The pharmacological treatment includes acetaminophen, indomethacin, and ibuprofen. For surgical treatment, the network includes surgical ligation, mini-thoracotomy, VATS, and interventional therapy. Finally, a network of pharmacological and surgical treatment will be created if possible.

### Statistical analysis and data synthesis

#### Pairwise meta-analysis

We will use Review Manager 5.3 (The Nordic Cochrane Centre, The Cochrane Collaboration, Copenhagen, Denmark) statistical analysis software for pairwise meta‐analysis. For dichotomous outcomes, the combined results will be calculated as odds ratios (ORs) and 95% confidence intervals (CIs). For continuous outcomes, we will calculate and meta-analyze mean differences (MD) or standardized mean differences (SMD) and their 95% CIs. We will use the Cochran’s *Q*-test and *I*^2^ statistics to assess and report heterogeneity. When *I*^2^ ≤ 40%, the heterogeneity is considered acceptable, and the fixed-effects model is used for the combined analysis. If *I*^2^ > 40%, the heterogeneity is considered to be large, and the source of heterogeneity will be explored. Sensitivity analysis, subgroup analysis, and meta-regression will be used to find the source of heterogeneity. Meanwhile, the clinical and methodological heterogeneity across studies will be reassessed. If no proper reasons will be found for high heterogeneity, the random effects model will be used to incorporate the heterogeneity. *P* < 0.05 was considered as statistically significant.

#### Network meta-analysis

Network meta-analysis will be conducted using a Bayesian Markov chain Monte Carlo (MCMC) framework. We will use the generalized linear modelling framework for network meta-analysis of randomized controlled trials. The convergence will be assessed using the Brooks-Gelman-Rubin (BGR) plots method. Both random and fixed models will be used. And the best model will be chosen based on the deviance information criterion (DIC) values. The model with lowest DIC value is usually selected because it is considered in most cases to be the most reliable. Transitivity is a key assumption of NMA and refers to the belief that an indirect comparison is a valid estimate of the unobserved direct comparison [30, 31]. The node splitting method will be used to examine the transitivity between direct and indirect comparisons if a loop connecting three or more arms exist [32]. If node-splitting analysis determined *P* < 0.05, the inconsistency model will be used for pooled analysis. Meanwhile, the consistency model will be used [33, 34]. The network meta-analysis results using inconsistency and consistency model will be compared. If the results using inconsistence and consistence model are not compared or there are any inconsistences between direct and indirect results, subgroup analysis, sensitivity analysis, or meta-regression will be used to find the source of inconsistence will be also used. Besides, we will rank all included interventions in terms of probability. The rank and probabilities of each intervention will be shown using surface under the cumulative ranking curve (SUCRA) and ranking plots (rank probability — rank curve). SUCRA is a numeric presentation of the overall ranking and presents a single number, ranging from 0 to 1, associated with each treatment [35]. If the SUCRA value of a certain intervention is close to 1, it is always ranked first, and if it is close to 0, it is always ranked last [36]. The MCMC simulation will be implemented using the R software (gemtc and pcnetmeta packages) (V.4.1.0) (http://cran.r-project.org/) with interfacing to OpenBUGS (V.3.2.3, MRC Biostatistics Unit, Cambridge, UK) (http://www.openbugs.net/w/Downloads) [37–39].

#### Subgroup analysis

According to the instrument to assess the Credibility of Effect Modification Analyses (ICEMAN) tool [40], subgroup analyses will be performed according to the following factors:

1. Participant characteristics (e.g., sex, age)
2. Study-specific factors are as follows:
  (a) Sample size
  (b) Overall study quality
  (c) Risk of bias (e.g., all studies versus low bias studies only)

#### Assessment of publication bias

The funnel plot and Egger test will be conducted to detect publication bias if the number of studies was more than ten.

#### Quality of evidence

We will assess the quality of the evidence by following the steps to assess the quality of treatment effect estimates for network meta-analysis as per the Grading of Recommendations Assessment, Development, and Evaluation (GRADE) [41] approach. The GRADE approach will assess five domains including study limitations, consistency of effect, imprecision, indirectness, and publication bias to assess the quality of the body of evidence for each outcome. We will use the following four steps to assess the quality of treatment effect estimates from NMA. Firstly, present direct and indirect treatment estimates for each comparison of the evidence network. Secondly, rate the quality of each direct and indirect effect estimate. In addition, present the NMA estimate for each comparison of the evidence network. Meanwhile, conceptual advances will be applied ((1) consideration of imprecision is not necessary when rating the direct and indirect estimates to inform the rating of NMA estimates, (2) there is no need to rate the indirect evidence when the certainty of the direct evidence is high and the contribution of the direct evidence to the network estimate is at least as great as that of the indirect evidence, (3) not trust a statistical test of global incoherence of the network to assess incoherence at the pairwise comparison level) [42]. Finally, rate the quality of each NMA effect estimate. It is classified into four levels: high level, moderate level, low level, and very low level.

### Sensitivity analysis

We will perform a sensitivity analysis to verify the robustness of the study results. This will be achieved by assessing the impact of the sample size, high risk of bias, missing data, and selected models. Following the analyses, if the quality of a study is judged to be low, it will be removed to ensure the robustness of the results.

### Ethics and dissemination

Ethical approvals and patient consent are not required because this is a network meta-analysis based on published trials. The findings of this project will provide a general review and evidence of the efficacy and safety of pharmacological treatment, surgical ligation, mini-thoracotomy, VATS, fluoroscopy-guided transfemoral venin and artery of percutaneous closure, fluoroscopy combined with TTE-guided transfemoral venin and artery of percutaneous closure, TTE-guided transfemoral artery of percutaneous closure, and TTE-guided transfemoral vein of percutaneous closure for PDA in children. The results will be submitted to a peer-reviewed journal for publication. We hope that these findings will help clinicians and patients choose a more appropriate repair method for PDA.

## Supplementary Information

**Additional file 1:** PRISMA-P (Different surgical procedures for the treatment of patent ductus arteriosus in children: a protocol for a network meta-analysis*

## Data Availability

Data sharing is not applicable as no datasets generated and/or analyzed for this study.

## References

[1] Marelli AJ, Ionescu-Ittu R, Mackie AS, Guo L, Dendukuri N, Kaouache M (2014). Lifetime prevalence of congenital heart disease in the general population from 2000 to 2010. Circulation.

[2] Anilkumar M (2013). Patent ductus arteriosus. Cardiol Clin.

[3] Schneider DJ, Moore JW (2006). Patent ductus arteriosus. Circulation.

[4] Ivey KN, Srivastava D (2006). The paradoxical patent ductus arteriosus. J Clin Invest.

[5] Kanabar K, Bootla D, Kaur N (2020). Outcomes of transcatheter closure of patent ductus arteriosus with the off-label use of large occluders (≥16 mm). Indian Heart J.

[6] Francis E, Singhi AK, Lakshmivenkateshaiah S, Kumar RK (2010). Transcatheter occlusion of patent ductus arteriosus in pre-term infants. JACC Cardiovasc Interv.

[7] Vidavalur R. Efficacy and Costs of Three Pharmacotherapies for Patent Ductus Arteriosus Closure in Premature Infants. Paediatr Drugs. 2022;24(2):93-102. 10.1007/s40272-022-00495-1 35229248

[8] Yanagi RM, Wilson A, Newfeld EA, Aziz KU, Hunt CE (1981). Indomethacin treatment for symptomatic patent ductus arteriosus: a double-blind control study. Pediatrics.

[9] Gersony WM, Peckham GJ, Ellison RC, Miettinen OS, Nadas AS (1983). Effects of indomethacin in premature infants with patent ductus arteriosus: results of a national collaborative study. J Pediatr.

[10] Shah SS, Ohlsson A. Ibuprofen for the prevention of patent ductus arteriosus in preterm and/or low birth weight infants. Cochrane Database Syst Rev. 2003(2):Cd004213. 10.1002/14651858.CD004213 12804505

[11] Erdeve O, Yurttutan S, Altug N (2012). Oral versus intravenous ibuprofen for patent ductus arteriosus closure: a randomised controlled trial in extremely low birthweight infants. Arch Dis Child Fetal Neonatal Ed.

[12] Al-Shaibi S, Abushanab D, Alhersh E, Kaddoura R, Pallivalappila AR, Al-Badriyeh D (2021). Use of ibuprofen for the closure of patent ductus arteriosus in preterm infants: a systematic review of meta-analyses. J Comp Eff Res.

[13] Alexi-Meskishvili VV, Böttcher W (2010). The first closure of the persistent ductus arteriosus. Ann Thorac Surg.

[14] Gross RE, Hubbard JP (1984). Landmark article Feb 25, 1939: Surgical ligation of a patent ductus arteriosus. Report of first successful case. By Robert E. Gross and John P. Hubbard. JAMA.

[15] Pavlek LR, Slaughter JL, Berman DP, Backes CH (2018). Catheter-based closure of the patent ductus arteriosus in lower weight infants. Semin Perinatol.

[16] Zahn EM, Nevin P, Simmons C, Garg R (2015). A novel technique for transcatheter patent ductus arteriosus closure in extremely preterm infants using commercially available technology. Catheter Cardiovasc Interv.

[17] Almeida-Jones M, Tang NY, Reddy A, Zahn E (2019). Overview of transcatheter patent ductus arteriosus closure in preterm infants. Congenit Heart Dis.

[18] Lenoir M, Wanert C, Bonnet D, et al. Anterior Minithoracotomy vs. Transcatheter Closure of Patent Ductus Arteriosus in Very Preterm Infants. Front Pediatr. 2021;9:700284. 10.3389/fped.2021.700284 PMC8640484 34869092

[19] Karaci AR, Sasmazel A, Turkay S (2013). Closure of a patent ductus arteriosus in pre-term neonates using a left anterior mini-thoracotomy. J Card Surg.

[20] Laborde F, Noirhomme P, Karam J, Batisse A, Bourel P, Saint MO (1993). A new video-assisted thoracoscopic surgical technique for interruption of patient ductus arteriosus in infants and children. J Thorac Cardiovasc Surg.

[21] Garcia AV, Lukish J (2017). Minimally invasive patent ductus arteriosus ligation. Clin Perinatol.

[22] Chen H, Weng G, Chen Z (2011). Comparison of posterolateral thoracotomy and video-assisted thoracoscopic clipping for the treatment of patent ductus arteriosus in neonates and infants. Pediatr Cardiol.

[23] Porstmann W, Wierny L, Warnke H (1967). Closure of persistent ductus arteriosus without thoracotomy. Ger Med Mon.

[24] Wang K, Pan X, Tang Q, Pang Y (2014). Catheterization therapy vs surgical closure in pediatric patients with patent ductus arteriosus: a meta-analysis. Clin Cardiol.

[25] Susheel Kumar TK (2019). Surgical management of patent ductus arteriosus. Congenit Heart Dis.

[26] Johnson JN, Sathanandam S, Naik R, Philip R (2019). Echocardiographic guidance for transcatheter patent ductus arteriosus closure in extremely low birth weight infants. Congenit Heart Dis.

[27] Shamseer L, Moher D, Clarke M (2015). Preferred Reporting Items for Systematic Review and Meta-Analysis Protocols (PRISMA-P) 2015: elaboration and explanation. BMJ.

[28] Hutton B, Salanti G, Caldwell DM (2015). The PRISMA extension statement for reporting of systematic reviews incorporating network meta-analyses of health care interventions: checklist and explanations. Ann Intern Med.

[29] Higgins JPT, Altman DG, Gøtzsche PC (2011). The Cochrane Collaboration's tool for assessing risk of bias in randomised trials. BMJ.

[30] Doyle F, Freedland K, Carney R (2019). Network meta-analysis of randomised trials of pharmacological, psychotherapeutic, exercise and collaborative care interventions for depressive symptoms in patients with coronary artery disease: hybrid systematic review of systematic reviews protocol. Syst Rev.

[31] Salanti G (2012). Indirect and mixed-treatment comparison, network, or multiple-treatments meta-analysis: many names, many benefits, many concerns for the next generation evidence synthesis tool. Res Synth Methods.

[32] Lu G, Ades AE (2004). Combination of direct and indirect evidence in mixed treatment comparisons. Stat Med.

[33] Yi K, Guo X, You T (2019). Standard median sternotomy, right minithoracotomy, totally thoracoscopic surgery, percutaneous closure, and transthoracic closure for atrial septal defects in children: a protocol for a network meta-analysis. Medicine (Baltimore).

[34] Dias S, Welton NJ, Caldwell DM, Ades AE (2010). Checking consistency in mixed treatment comparison meta-analysis. Stat Med.

[35] Marotta N, Demeco A, Moggio L (2020). Comparative effectiveness of breathing exercises in patients with chronic obstructive pulmonary disease. Complement Ther Clin Pract.

[36] Salanti G, Ades AE, Ioannidis JPA (2011). Graphical methods and numerical summaries for presenting results from multiple-treatment meta-analysis: an overview and tutorial. J Clin Epidemiol.

[37] Neupane B, Richer D, Bonner AJ, Kibret T, Beyene J (2014). Network meta-analysis using R: a review of currently available automated packages. PLoS ONE.

[38] Zhang J, Carlin BP, Neaton JD, et al. Network meta-analysis of randomized clinical trials: reporting the proper summaries. Clinical trials (London, England). 2014;11(2):246-262. 10.1177/1740774513498322 PMC3972291 24096635

[39] Shim SR, Kim SJ, Lee J, Rücker G. Network meta-analysis: application and practice using R software. Epidemiology and health. 2019;41:e2019013. 10.4178/epih.e2019013 PMC6635665 30999733

[40] Schandelmaier S, Briel M, Varadhan R (2020). Development of the Instrument to assess the Credibility of Effect Modification Analyses (ICEMAN) in randomized controlled trials and meta-analyses. CMAJ.

[41] Puhan MA, Schünemann HJ, Murad MH (2014). A GRADE Working Group approach for rating the quality of treatment effect estimates from network meta-analysis. BMJ.

[42] Brignardello-Petersen R, Bonner A, Alexander PE (2018). Advances in the GRADE approach to rate the certainty in estimates from a network meta-analysis. J Clin Epidemiol.

